# Descriptive Analysis of Articles and Advertisements Pertaining to Skin Cancer Prevention in 2 Popular US Parenting Magazines, 2000–2010

**DOI:** 10.5888/pcd10.120200

**Published:** 2013-04-04

**Authors:** Corey H. Basch, Grace Clarke Hillyer, Charles E. Basch

**Affiliations:** Author Affiliations: Grace Clarke Hillyer, Charles E. Basch, Columbia University, New York, New York.

## Abstract

Magazines focused on parenting are popular in the United States, and parents may use them to guide decisions about the health of their children. We analyzed issues of 2 popular parenting magazines published in the past 11 years during the months of peak ultraviolet radiation exposure for content related to sun protection and for advertisements for skin products that did and did not contain sun protection factor. Only 24 of 2,594 articles addressed the topic of sun protection for skin cancer prevention. Although advertising is pervasive in these magazines, the extent to which such advertising focuses on products with sun protection factor was low. These findings suggest that parenting magazines can do more to assist parents in making informed decisions about preventing skin cancer risk among youth.

## Objective

Skin cancer is the most common form of cancer in the United States (1). Excessive exposure to ultraviolet radiation at a young age contributes to the risk of developing melanoma later in life (2). Parents play a crucial role in minimizing their children’s excessive exposure to ultraviolet radiation and in instilling life-long protective habits (3–5).

*Parents* and *Parenting* are the 2 most popular US magazines that target parents, comprising more than 4 million subscriptions and a combined readership of approximately 24 million in 2011 (6). We assessed a sample of these publications to describe the prevalence of articles and advertisements related to skin cancer prevention.

## Methods

We used direct observation to identify articles and advertisements related to skin, skin care, and health in the spring and summer issues (May, June, July, and August) of *Parents* and *Parenting* magazines during an 11-year period (2000–2010). Because the magazines had to be viewed in their entirety, only fully intact, hard copies of magazines or complete versions of microfilm could be used. Articles related to skin, skin care, and health were identified from the table of contents and examined. Each article was read, and word counts were conducted on all articles; content was assessed in terms of the type of information that each article contained. Articles about skin were grouped into 3 categories: “sun protection,” “beauty-related,” and “other” (eg, moles, rashes). During the coding process, we used a coding sheet for each issue that had space to record content, word count, and the types of advertisements contained in the issue.

The total number and type of all advertisements were recorded by reviewing each page of the magazine. Special notation was made for advertisements related to sunless tanning products, sun block, and skin products, both with and without sun protection factor (SPF). Although many categories of advertisements were related to varied products and services, we report only those related to skin and health in general. Health advertisements were coded as “adult health” (eg, birth control), “child health” (eg, medication for attention-deficit/hyperactivity disorder), and “miscellaneous health,” which was not aimed specifically at adults or children (eg, dental care products). We examined the frequency of articles and advertisements devoted to skin and health relative to all articles and advertisements. Ranges for each item were also calculated.

## Results

Of the 2,594 articles reviewed (range, 181–290 per year observed), 57 (2.2%) were related to skin and 509 (19.6%) were related to health (Table). Of the skin-related articles, 24 (42.1%) focused on sun protection: the avoidance of sun during peak hours, seeking shade, wearing protective clothing and eyewear, and use of sun block. Health-related articles covered varied topics from fitness and nutrition to emotional and behavioral issues. The frequency and percentage of articles related to health in general and skin, in particular, were similar in both magazines. No substantive changes in the frequency of skin articles were observed over time (for both magazines, range per year for sun protection, 1–4).

**Table:** Total Articles (N = 2,594)^a^ and Advertisements (N = 6,307)^b^, by Type, in Spring and Summer Issues of *Parents* and *Parenting* Magazines (May, June, July, and August), 2000–2010

Type	n (%)	Range Per Year
**Articles**		
**Skin-related**	57 (2.2)	2–7
Sun protection	24 (42.1)	1–4
Beauty-related	13 (22.8)	0–3
Other	16 (28.1)	0–5
**Health-related**	509 (19.6)	25–61
**Advertisements**		
**Skin products**	538 (8.5)	21–79
Sunless tanners	4 (0.7)	0–3
Sun block	64 (11.9)	0–16
Skin products with SPF	30 (5.6)	0–5
Skin products without SPF	440 (81.8)	18–71
**Health-related**	844 (13.4)	55–110
Adult health	280 (33.2)	16–38
Child health	429 (50.8)	24–55
Miscellaneous	135 (16.0)	0–24
**Other**	4,925 (78.1)	355–533

Abbreviation: SPF, sun protection factor.

a Range per year, 181–290.

b Range per year, 449–716.

We identified 6,307 (range, 449–716 per year observed) advertisements in both magazines during the observation period. Nearly 4 of 5 (78.1%) pertained to topics that were not related to health (eg, cleaning products, department stores, food and beverages, vacation destinations). Among the remaining 1,382 advertisements, 538 (8.5%) focused on skin products, but most (n = 440, 81.8%) pertained to skin products without SPF, particularly beauty products such as moisturizer and make-up foundation. A total of 64 advertisements were for sun block, representing approximately 1% of total ads. Only 30 ads were identified for skin products with SPF that were not sun block. Of the 844 health-related advertisements, more than half were related to children’s health (n = 429, 50.8%), 280 to adult health (33.2%), and 135 (16.0%) to miscellaneous health-related items. The nature and scope of advertising regarding skin cancer risk reduction was very similar in the 2 magazines during the 11-year period.

## Discussion

Of the most popular parenting magazines in the United States, little attention is given during the spring and summer issues to sun protection in articles and advertisements. The magazines were reviewed during the months of peak ultraviolet exposure, but only 24 of 2,594 articles (<1%) during an 11-year period addressed the topic of sun protection for skin cancer prevention. Furthermore, although advertising in the magazines is pervasive, the extent to which such advertising focuses on products with SPF during the months when ultraviolet radiation is most damaging and dangerous was low.

The etiology of skin cancer is well understood and indicates that overexposure to ultraviolet radiation early in life is a preventable risk factor (3). Studies indicate the need to reduce children’s overexposure to ultraviolet radiation from the sun (3,4) and that behaviors that increase risk of skin cancer are prevalent among adolescents (7–9). Parents are responsible for protecting young children from sunburns and less extreme overexposure to ultraviolet radiation as well as for educating them to minimize skin cancer risk behaviors.

Americans are exposed to a substantial amount of information via varied media channels every day. With changes in the coverage of health in the media, the context in which parents obtain health information has changed dramatically. Magazines that target parents can help parents make informed decisions about reducing their children’s skin cancer risks in childhood and adolescence. The 2 magazines we reviewed reportedly have approximately 24 million readers (6), who in turn may influence far more children (10,11). These findings suggest that parenting magazines and companies advertising in this medium can do much more to assist parents in making informed decisions about preventing skin cancer risk among youth.
